# Classification and Identification of Bacteria by Mass Spectrometry and Computational Analysis

**DOI:** 10.1371/journal.pone.0002843

**Published:** 2008-07-30

**Authors:** Sascha Sauer, Anja Freiwald, Thomas Maier, Michael Kube, Richard Reinhardt, Markus Kostrzewa, Klaus Geider

**Affiliations:** 1 Max Planck Institute for Molecular Genetics, Berlin, Germany; 2 Bruker Daltonik, Leipzig, Germany; 3 Julius Kuehn Institute for Plant Protection in Fruit Crops and Viticulture, Dossenheim, Germany; University of Wisconsin-Milwaukee, United States of America

## Abstract

**Background:**

In general, the definite determination of bacterial species is a tedious process and requires extensive manual labour. Novel technologies for bacterial detection and analysis can therefore help microbiologists in minimising their efforts in developing a number of microbiological applications.

**Methodology:**

We present a robust, standardized procedure for automated bacterial analysis that is based on the detection of patterns of protein masses by MALDI mass spectrometry. We particularly applied the approach for classifying and identifying strains in species of the genus *Erwinia.* Many species of this genus are associated with disastrous plant diseases such as fire blight. Using our experimental procedure, we created a general bacterial mass spectra database that currently contains 2800 entries of bacteria of different genera. This database will be steadily expanded. To support users with a feasible analytical method, we developed and tested comprehensive software tools that are demonstrated herein. Furthermore, to gain additional analytical accuracy and reliability in the analysis we used genotyping of single nucleotide polymorphisms by mass spectrometry to unambiguously determine closely related strains that are difficult to distinguish by only relying on protein mass pattern detection.

**Conclusions:**

With the method for bacterial analysis, we could identify fire blight pathogens from a variety of biological sources. The method can be used for a number of additional bacterial genera. Moreover, the mass spectrometry approach presented allows the integration of data from different biological levels such as the genome and the proteome.

## Introduction

In general, new technologies for accurate and rapid identification of bacteria are essential to epidemiological surveillance, i.e. the recognition of early outbreak, the analysis of cross-transmission, and the monitoring of treatment programs including application of antagonistic bacteria. For classifying and identifying bacterial species, cumbersome physiological, serological, biochemical, chemotaxonomic, and more recently genomic methods have been routinely applied in microbiology [1]. For example, genetic approaches using digital genomic information for the detection of 16S rRNA genes provide specific tools for classification of bacteria. The analysis of DNA sequence similarities of housekeeping genes is also being used for multilocus sequence typing (MLST) approaches [2], which can however be tedious in mass screening. PCR-based methods to detect pathogens are available but cannot be used for classification, especially in the case of unknown bacterial samples. Moreover, the analysis of bacteria such as the highly conserved fire blight pathogen *E. amylovora* requires special efforts to differentiate strains by pulsed field gel electrophoresis analysis or by sequencing of virulence genes [3].

Mass spectrometric approaches that use molecular biological sample preparation have been shown recently in microbial typing [4], [5]. These methods comprise highly sophisticated instrumentation, and the associated costs from sample preparation make the operation prohibitive for general use. Alternatively, MALDI time-of-flight (TOF) mass spectrometry protein profiling of whole bacterial cells can be applied for detection of bacteria [6]. However, most of these procedures have so far not exceeded proof-of-principle level and were applied only to a limited number of bacterial species [7], [8]. Moreover, all these procedures do not have proven maturity for easy and systematic application in microbiology. Consequently, biologists have not consistently utilized these approaches despite their great potential.

In the exemplary study of this article we focused on the mass spectrometry analysis of bacteria of the genus *Erwinia* and related (phytopathogenic) bacteria. The genus *Erwinia* comprises several bacterial species, many of them connected to plant diseases [9]. The *Erwinia* species belong to the family of *Enterobacteriaceae*, which also include *Escherichia coli*, *Yersinia* spp., *Shigella* spp., and *Salmonella* spp. *Erwinia amylovora* causes the devastating fire blight disease of rosaceous plants, such as apple and pear trees and some ornamentals. Since the last century, outbreaks of this disease have caused economical crisis in agriculture [10].

## Results

**Figure 1:**
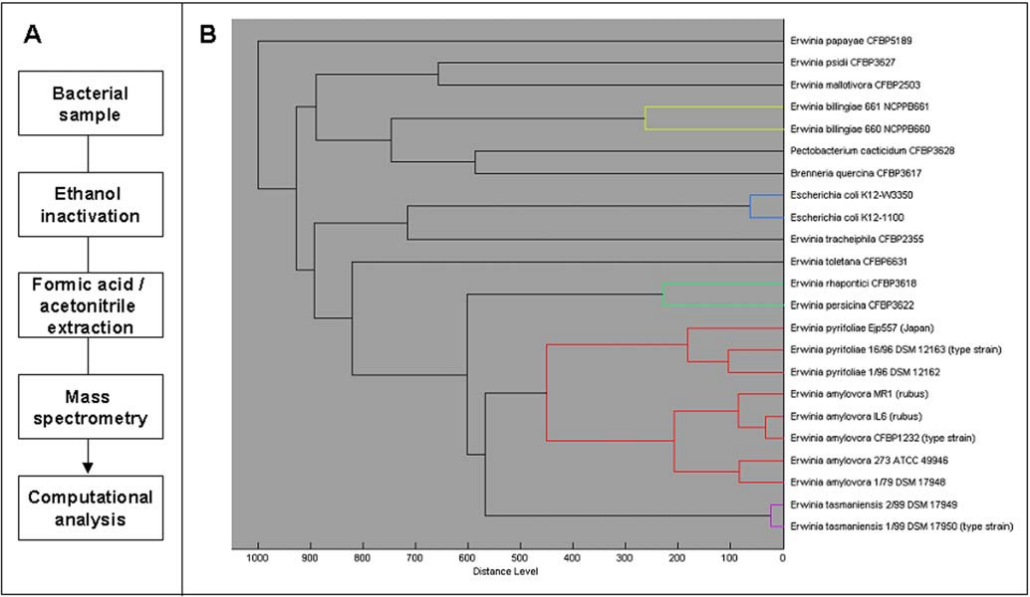
A: A general scheme of the procedure. Bacterial colonies are subjected to chemical treatment. Samples can be analyzed within a few minutes by MALDI mass spectrometry and mass spectra are transferred to analysis and identification software. B: Classification of bacteria. Based on the protein mass patterns, bacterial strains can be clustered hierarchically. A dendrogram generated by this approach including a comprehensive set of *Erwinia* type strains was displayed. Species with distance levels over 500 had completely different mass signal patterns. Comparison of spectra of these species for a distance measure was thus uninformative. Strains clustering with distance levels lower than 500 could be classified up to the species and partially to subspecies level. The limit of resolution was set by the distances derived from measurement variability.

We describe a standardized sample preparation and analytical procedure for easy bacterial classification and identification by MALDI mass spectrometry detection of protein mass patterns (Figure 1A). This method includes the use of advanced bioinformatics analysis and a database resource containing a comprehensive number of bacterial reference mass spectra. We enlarged the potential of this approach by genotyping an informative single nucleotide polymorphism (SNP) by mass spectrometry.

All *Erwinia* bacteria analyzed in this study were conventionally cultured in liquid medium. To fulfill biological safety requirements the potentially pathogenic *Erwinia* samples were inactivated efficiently in ∼73% ethanol. This treatment completely destroyed the viability of the bacteria after an hour of fixation. Once the samples were fixed, they were treated with formic acid for cell wall disruption and acetonitrile for protein extraction (Materials and Methods). Finally, a fraction of the protein samples was prepared on a MALDI target plate and mass spectra accumulation was performed automatically.

Applying the standardized experimental procedure including culturing in liquid media (Materials and Methods), we generated a reference mass spectra database containing main spectra libraries (MSPs) of bacteria of the genus *Erwinia* and some other related bacteria. Therefore, we accumulated twenty mass spectra to achieve above average quality spectra. Protein mass patterns were detected in the mass range of 2,000 to 20,000 Da. The building of a general database of reference mass spectra, which are produced by the standardized protocol applied in this study, is currently underway. To date, this database comprises more than 2800 bacterial strains, including the genera *Pantoea*, *Shigella*, *Listeria*, *Salmonella*, and *Klebsiella* (Materials and Methods). The database has been implemented in our analysis software (Materials and Methods) and was used for the identification experiments shown below. To reproduce the results of this study and to test the software for additional applications, the software package and the reference mass spectra are freely available as a CD that can be requested from the authors.

**Figure 2:**
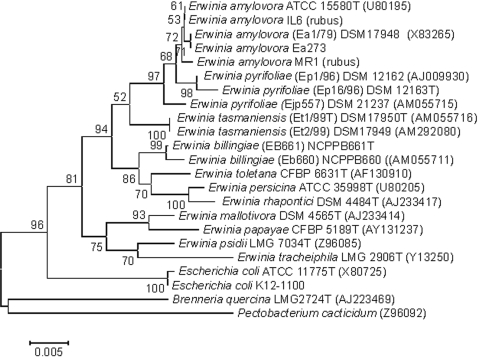
Phylogenetic analysis. For comparison to mass spectrometry based clustering a conventional 16S rRNA sequence-based dendrogram generated with ClustalW and Mega 3.1 is shown. Phylogenetic distances were estimated by the method of Jukes and Cantor [15]. The tree topology was inferred by neighbor-joining method with a bootstrap value of 1000. For the reconstruction of phylogeny, the neighbor-joining and maximum-parsimony procedures produced similar results. The 16S rRNA sequences of *E. coli* strains were identical to the *E. coli* strains shown in the dendrogram generated on the basis of mass spectra.

**Figure 3:**
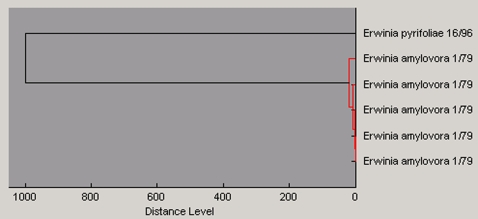
Determination of experimental variation of the procedure. *E. amylovora* sub-species 1/79 (German strain) was grown in LB-glucose in quintuplicate and analyzed. *E. pyrifoliae* 16/96 was used as an outlier for clustering. Distance levels below ∼30–50 could not be resolved any further by our clustering approach due to experimental noise. The experimental variation was presumably caused mainly by slight changes in matrix-protein co-crystallization such as room temperature, pressure, and humidity. In this exemplary experiment, the coefficient of variation (CV) for intra run was 0.31 and for inter run 0.35. Similar CVs were observed for the identification experiments presented in the paper. As shown in Table 1, the robust analysis algorithms applied can easily handle the experimental variation that is associated with our approach.

For phylogenetic analysis, we clustered hierarchically mass spectra of type strains and others in dendrograms according to their mass signals and intensities (Figure 1B). Each reference spectrum of a dataset was compared with the other reference spectra, thus resulting in a matrix of cross-wise identification values. This matrix is used to calculate the distance values for each pair. Based on these distance values the dendrogram was generated using the according function of the statistical toolbox of Matlab 7.1 (The MathWorks Inc., USA), which was integrated in the analysis software (Materials and Methods). The clustering approach applied was based on similarity scores implemented in the analysis software. For comparison, a phylogenetic dendrogram was generated from 16S rRNA sequences (Figure 2). Interestingly, the mass spectrometry and the sequence-based dendrograms showed similar clustering within related species. As for the other cell parameters, co-evolution of 16S rRNA sequences and ribosomal proteins, which are mainly detected by mass spectrometry from whole bacterial cells, could be assumed [11]. Thus, the mass spectrometry-based dendrogram made significant sense from a biological point of view. As for 16S rRNA, related species such as *E*. *amylovora* and *E*. *pyrifoliae* from Korea and Japan or epiphytic bacteria like *E. persicina* and *E. rhapontici* clustered closely together. This observation was confirmed by data generated from housekeeping genes, as well as microbiological and biochemical studies [12]. *E. tasmaniensis* was linked to *E. amylovora* and to *E. pyrifoliae*, whereas *E. billingiae* was placed on a different branch. The two exemplary *E. tasmaniensis* strains from different isolation origins clustered closely together and their distance based on comparison of protein mass patterns were within the range of experimental noise of the procedure (Figure 1B). The average reproducibility of the procedure is exemplarily documented in Figure 3. In general, we observed coefficient of variation (CV) values slightly above 0.3 in intra- and inter-run experiments.

**Figure 4:**
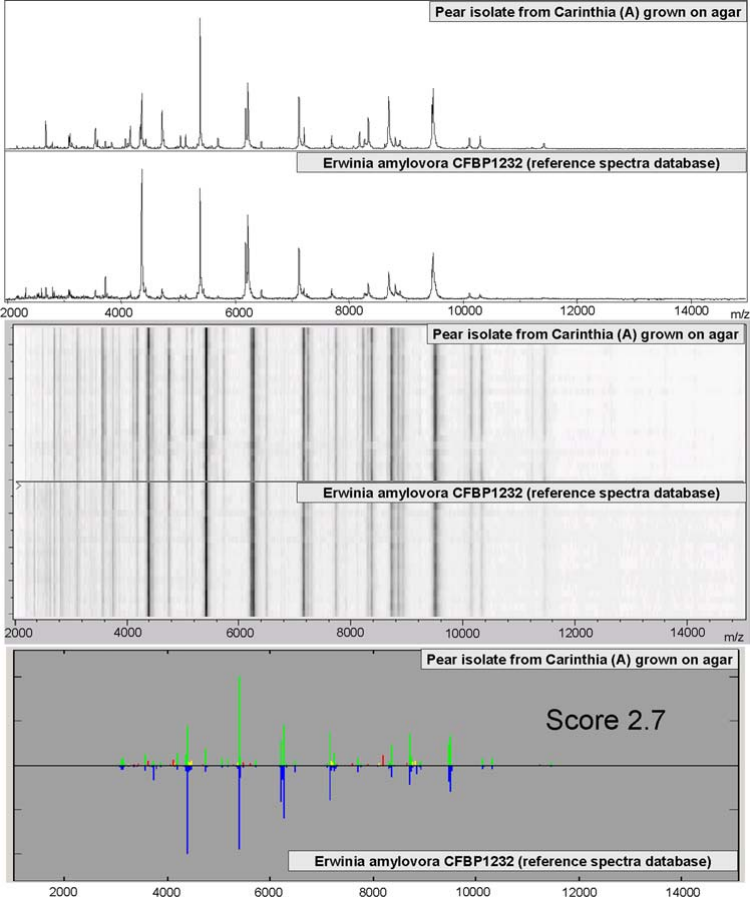
Identification of *E. amylovora* from diseased pears. A typical mass spectrum of a bacterial sample taken from necrotic wood compared with a matching spectrum from the reference library. (Top) Original mass spectra, (Middle) respective pseudo gel-view showing a bar-code of masses and their intensities, (Bottom) identification by comparison of experimental and reference mass spectra using a pattern matching algorithm. In this example, a highly reliable identification score of 2.7 was obtained from the identification of *E. amylovora* (CFBP1232).

With the entire reference spectra library, we could identify unambiguously bacteria of the genus *Erwinia*. Therefore, we analyzed a number of isolates from different locations of the world and samples from necrotic wood of diseased pear trees from Carinthia. In many cases, plant samples contained mixtures of bacteria of the genus *Erwinia* or other genera. Conventional culturing on agar and/or in liquid media prior to chemical treatment was performed to produce sufficient amount and homogeneity of bacterial samples for the analysis. The approach became robust against growth times once the bacteria have entered the stationary phase (data not shown). For initial species identification of the different isolates listed in Table 1, we applied a pattern-matching algorithm, which calculated calibrated m/z values, average intensities, and frequency distributions of each mass signal in different measurements. With our approach, an identification score of 2.0 or higher indicated a reliable identification of species. As summarized in Table 1, we identified unambiguously pathogenic bacteria such as *E*. *rhapontici, E*. *persicina, E*. *amylovora,* and *Brenneria quercinia* (syn. *Erwinia quercinia*) from a variety of plant samples. Figure 4 shows a typical result of an identification experiment performed in this study. Although the bacteria were grown on different media, similar identification scores were obtained due to their almost identical mass spectra (Table 1). We could even detect the fire blight pathogen in washes of plant tissue from *in vitro* propagated pears that were infected with about 10^7^ cells of *E. amylovora*. In a larger study, we additionally analyzed over several months a comprehensive number of isolates (Table 1). We screened successfully the mass spectra of these isolates with the entire reference spectra database. In all cases we correctly detected the respective samples with similar identification scores. All mass spectrometry-based identification results presented in this paper were consistent with 16S rRNA sequencing and microbiological and biochemical data derived from intensively studied isolates that were stored in our laboratory.

**Table 1:** Summary of the identification scores produced by the pattern-matching algorithm.

Isolates	Preparation procedure	Identified microorganism	ID score	Specific mass signals (m/z)
Carinthia (A)	agar	*E.billingiae* 661	2.5	
Carinthia (A)	LBglc	*E.billingiae* 661	2.6	
Carinthia (A)	LBglc	*E.billingiae* 661	2.7	
Australia	LBglc	*E.tasmaniensis* 1/99	2.7	
Australia	LBglc	*E.tasmaniensis* 1/99	2.5	
South Africa	LBglc	*E.tasmaniensis* 1/99	2.8	
South Africa	LBglc	*E.tasmaniensis* 1/99	2.8	
Heidelberg (D)	LBglc	*E.tasmaniensis* 1/99	2.5	
Carinthia (A)	agar	*E.quercini*	2.4	
Austria	LBglc	*E.persicina*	2.3	9472, 9513
Carinthia (A)	LBglc	*E.rhapontici*	2.3	4392, 7680, 8241, 9457
Carinthia (A)	LBglc	*E.rhapontici*	2.3	4392, 7680, 8241, 9457, 9483
Canada	LBglc	*E.amylovora* MR-1	2.2	4092, 8186, 5561
Canada	LBglc	*E.amylovora* MR-1	2.1	4092, 8186, 5561
South Korea (DSM 12393)	LBglc	*E.pyrifoliae* 16/96	2.6	7696, 7235
South Korea (DSM 12394)	LBglc	*E.pyrifoliae* 16/96	2.5	7696, 7235
Korea	LBglc	*E.pyrifoliae* 16/96	2.6	7696, 7235
Japan	LBglc	*E.pyrifoliae* Ejp557	2.5	7696, 4722, 9445
Infected pear blossoms	directly	*E.amylovora* CFBP1232	2.4	8302, 8726, 8842, 9510
Infected pear blossoms	directly	*E.amylovora* CFBP1232	2.5	8302, 8726, 8842, 9510
Carinthia (A)	agar	*E.amylovora* CFBP1232	2.6	8302, 8726, 8842, 9510
Carinthia (A)	agar	*E.amylovora* CFBP1232	2.7	8302, 8726, 9510
Carinthia (A)	LBglc	*E.amylovora* CFBP1232	2.3	7594, 8725, 8842, 9510
England	LBglc	*E.amylovora* CFBP1232	2.6	8302, 8726, 8842, 9510
France	LBglc	*E.amylovora* CFBP1232	2.5	8302, 8726, 8842, 9510
Spain	LBglc	*E.amylovora* CFBP1232	2.6	8302, 8726, 8842, 9510
Hollande	LBglc	*E.amylovora* CFBP1232	2.5	8302, 8726, 9510
Germany	LBglc	*E.amylovora* CFBP1232	2.8	8302, 8726, 8842, 9510
Germany	LBglc	*E.amylovora* CFBP1232	2.6	8302, 8726, 8842, 9510
Stuttgart region (D)	LBglc	*E.amylovora* CFBP1232	2.7	8302, 8726, 8842, 9510
Stuttgart region (D)	LBglc	*E.amylovora* CFBP1232	2.7	8302, 8726, 8842, 9510
Bavaria (D)	LBglc	*E.amylovora* CFBP1232	2.7	8302, 8726, 8842, 9510
Switzerland	LBglc	*E.amylovora* CFBP1232	2.7	8302, 8726, 8842, 9510
Rogow (Pl)	LBglc	*E.amylovora* CFBP1232	2.6	8302, 8726, 8842, 9510
Mitilini (GR)	LBglc	*E.amylovora* CFBP1232	2.7	8302, 8726, 8842, 9510
Croatia	LBglc	*E.amylovora* CFBP1232	2.5	8302, 8726, 9510
Washington (USA)	LBglc	*E.amylovora* CFBP1232	2.5	8726, 9510
Ohio (U.S.A.)	LBglc	*E.amylovora* CFBP1232	2.4	8726, 8842, 9510
California (USA)	LBglc	*E.amylovora* CFBP1232	2.7	8302, 8726, 8842, 9510
Oregon (USA)	LBglc	*E.amylovora* CFBP1232	2.7	8302, 8726, 8842, 9510
Oregon (USA)	LBglc	*E.amylovora* CFBP1232	2.7	8302, 8726, 8842, 9510
Niagara region (CDN)	LBglc	*E.amylovora* CFBP1232	2.6	8302, 8726, 8842, 9510
Australia	LBglc	*E.amylovora* CFBP1232	2.7	8302, 8726, 8842, 9510
Australia	LBglc	*E.amylovora* CFBP1232	2.7	8302, 8726, 8842, 9510
Australia	LBglc	*E.amylovora* CFBP1232	2.7	8302, 8726, 8842, 9510
New Zealand	LBglc	*E.amylovora* CFBP1232	2.7	8302, 8726, 8842, 9510
New Zealand	LBglc	*E.amylovora* CFBP1232	2.7	8302, 8726, 8842, 9510
Israel	LBglc	*E.amylovora* CFBP1232	2.7	8302, 8726, 8842, 9510
Egypt	LBglc	*E.amylovora* CFBP1232	2.6	8302, 8726, 8842, 9510
Egypt	LBglc	*E.amylovora* CFBP1232	2.5	8302, 8726, 8842, 9510

**Figure 5:**
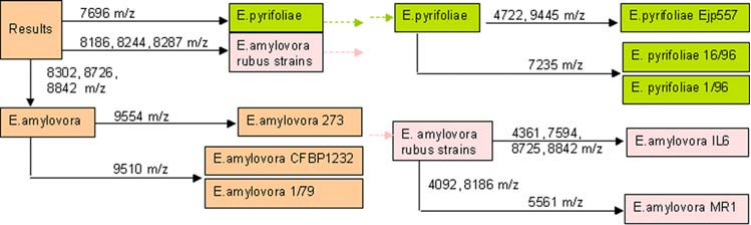
Weighted pattern matching. Starting from a given pattern matching result, which cannot differentiate between closely related sub-species, a limited number of mass signals are selected for accurate identification. The flow-chart shows the combination of selected specific mass signals to differ important *Erwinia* sub-species. For example, an initial pattern matching analysis revealed two potential candidate strains, *E. amylovora* and *E. pyrifoliae*. The mass signals at 7696 m/z and the mass peaks at 8186, 8244, 8287 m/z are specific for *E. pyrifoliae* and *E. amylovora* rubus strains, respectively, which distinguish them from *E. amylovora*. The mass peak at 9554 m/z distinguished the North American type strain *E. amylovora* 273 from the other *E. amylovora* strains of American and European origin, which instead have an additional peak at 9510 m/z. For the automated weighted pattern analysis, specific values listed in Table 1 were assigned to these marker masses. This approach was applied to distinguish closely related *Erwinia* strains as shown in Table 2.

We could identify a number of bacteria from different locations and deriving from different biological samples: bacteria directly taken from infected pears, isolated bacteria grown on agar, or bacteria additionally grown in liquid medium (LB-glucose). Log scores over 2 were considered reliable for type strain identification using the pattern-matching approach. These identification scores could be easily replicated with the same strains analyzed several times. Specific mass signals were used to accurately identify sub-species by visual inspection of mass spectra or by software-supported weighted pattern matching as demonstrated in Figure 5 and in Tables 2 and 3.

For accurate identification of closely related species such as the plant pathogens *E*. *amylovora* and *E*. *pyrifoliae*, we used a weighted pattern-matching algorithm. This algorithm uses selected characteristic mass signals to which specific values can be assigned in the analysis (Figure 5). As shown in Table 2, weighted pattern-matching helped to neatly determine very closely related strains that could not be distinguished by the initial pattern-matching procedure. The settings used in this study are summarized in Table 3.

**Table 2:** Weighted pattern-matching.

	MSP Analysis		Weighted Analysis	
Isolate	Matching MO	Score	Matching MO	Score
Infected pear	*Erwinia amylovora* IL6 (rubus)	2.5	*Erwinia amylovora* CFBP1232	2.4
blossoms	*Erwinia amylovora* CFBP1232	2.4	*Erwinia amylovora* IL6 (rubus)	1.7
(directly isolated)	*Erwinia amylovora* 1/79	2.3	*Erwinia pyrifoliae* 16/96	1.6

Weighted pattern-matching contributes to an increased accuracy of automated bacterial identification. For example, in the case of pears infected with *E. amylovora* (German strain Ea1/79), initial pattern matching analysis could not differentiate between different *E. amylovora* subspecies. Visual inspection of specific mass signals listed in Table 1 helped to unambiguously identify the correct sub-species. Software-supported weighted pattern matching leads to an accurate analysis of the correct subspecies, for example, the *E. amylovora* (type strain CFBP1232) was identified unambiguously. More information on the weighted pattern matching analysis is depicted in Figure 5 and in Table 3.

**Table 3:** Settings for weighted-pattern matching algorithm.

Species	E.a. 1232	E.a. 273	E.a. IL6	E.a. MR1	E.p. Ejp557	E.p. 16/96
Specific mass signal (m/z)						
3750	1000					
4361	1000	1000	1000			
8725	1000	1000	1000			
8842	1000	1000	1000			
9510	5000		1000			
9554		5000				
7594			2500			
8244			5000			
8287			5000	2000		
4092				5000		
5561				5000		
8186				5000		
7696					5000	5000
4722					5000	
9445					5000	
7235						5000

To differentiate between the various *E. amylovora* (E.a.) and *E. pyrifoliae* (E.p.) sub-species, a weighted-pattern matching algorithm as described in the Methods section was applied. For sub-species identification specific masses in the reference spectra that allow differentiation between sub-species are given an overemphasized value. For *E. amylovora* unspecific mass signals were set to zero except for the mass signals listed in the table. *E. pyrifoliae* sub-species can be determined by changing the weight values for the mass signals given in the Table. By summation of all these values each entry in the library acquires a specific number.

**Figure 6:**
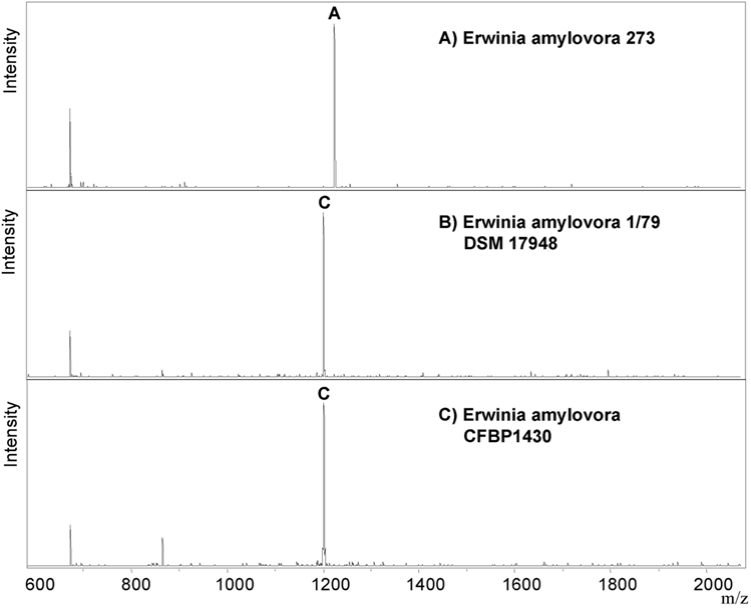
SNP genoytyping. We genotyped a SNP in the *galE* gene by MALDI-MS to distinguish *E. amylovora* strains of North American origin (*E. amylovora* 273) from that of European/Mediterranean origin such as *E. amylovora* 1/79 (DSM 17948) and CFBP1430. Masses detected in the spectra correspond to an A-allele (theoretical mass: 1225 m/z) or a C-allele (theoretical mass: 1201 m/z).

The integration of data from different biological levels refines the analysis, which has so far rarely been exploited in bioanalytics [1]. As shown in Figure 6 and Table 4, MALDI mass spectrometry can be used for integrating the analysis of data derived from detection of protein mass patterns and genomic markers such as SNPs [13]. We made use of a novel SNP that we discovered by re-sequencing of the *galE* gene of several *E. amylovora* strains (Figure S1). Genotyping of this informative SNP by mass spectrometry [13] allows us to distinguish *E*. *amylovora* strains of North American from European origin, which was impossible by merely analyzing protein mass patterns.

**Table 4:** A short exemplary list of SNP genotypes of the *galE* gene detected by MALDI mass spectrometry.

Microorganism	*galE* gene SNP 175	Origin of isolates
*E. amylovora* 273 ATCC 49946	A	USA
*E. amylovora* isolate	A	Washington, USA
*E. amylovora* isolate	A	California, USA
*E. amylovora* isolate	A	Niagara region (CDN)
*E. amylovora* 1/79 DSM 17948	C	Germany
*E. amylovora* isolate	C	Stuttgart region (D)
*E. amylovora* CFBP1430	C	North of France
*E. amylovora* isolate	C	Egypt

The SNP distinguishes *E. amylovora* strains of North American origin from the European/Mediterranean origin. The reference sequences are deposited as Figure S1.

## Discussion

As compared to protein identification based mass spectrometry methods [14], our procedure is robust against slight mass deviations detected by the mass spectrometer. Moreover, in many cases in microbiology - such as for many species of *Erwinia* - complete genomic or protein sequence databases are not available, which inhibits the use of identification-based mass spectrometry approaches. In practice, such approaches could rarely generate additional information for bacterial identification to the fingerprinting method shown here. Mainly ribosomal proteins as in the case of *E. coli* and other *Enterobacteriaceae* would be identifed with enormous cost and time commitment.

Other recently published mass spectrometry procedures for microbial typing rely on the detection of fragments of nucleic acids [4], [5]. By using a template amplification procedure these approaches are potentially very sensitive. However, the analyte fragments were produced in a sequence of parallel molecular biological reactions, which significantly contribute to the costs of these procedures.

DNA sequencing is one of the gold standards for characterization of bacteria, but this approach cannot be easily applied for fast classification and identification. In general, DNA-based methods such as PCR require optimization for setting up specific assays for each bacterial strain. Once they are being set up, conventionally applied procedures such as real-time PCR might be more specific and faster for bacterial identification than our method. However, our procedure can be readily applied and does not require reference sequences. Moreover, culturing of bacteria on agar plates and later on in liquid cultures reduces the sample complexity and improves the control of environmental samples. As it is shown for several *Erwinia* species, our method is largely independent from culturing conditions. Even when minimal media have to be used, however, potential culture-dependency of some mass signals could be excluded from the software analysis.

The protein mass pattern detection approach presented in this study is independent from DNA sequence information and complementary to DNA sequencing or PCR-based approaches. As we have shown here, digital information encoded in informative regions of bacterial genomes, such as a single SNP, can provide additional resolution in the mass spectrometry data analysis [14]. Data integration from different biological levels as demonstrated in this paper has so far rarely been applied in bioanalytics. Particularly mass spectrometry allows integrating biological information as this technique can be used to detect both, nucleic acids and proteins. In general such an integrated approach would certainly improve the accuracy and reliability in many diagnostic life sciences applications.

Due to the specificity, speed of analysis and low costs for consumables, our method for classifying and identifying bacterial species can replace a number of conventionally applied but cumbersome physiological, serological, biochemical, and chemotaxonomic procedures. The standardized bacterial detection procedure presented herein is facile and reproducible. It fulfils biological safety requirements and can be easily scaled up. At least 10^7^ bacterial cells are required for our preparation procedure to generate reliable results, which can be easily produced by cell culturing. Excluding the time for bacterial culturing, our approach takes 90 minutes from sample preparation to identification.

The method presented requires only a simple time-of-flight (TOF) mass spectrometer. Due to the software support demonstrated in this study, the method can be used for bacterial identification in a variety of applications without (extensively) analyzing mass spectra. The procedure can be easily applied by a microbiologist with essential knowledge in mass spectrometry. To provide the basis for large-scale longitudinal evaluation studies that can undergo specific testing of a large variety of different bacteria, we will steadily increase the numbers of bacterial type strain reference spectra. Therefore, we will apply the procedure presented in conjunction with the use of analysis software and the mass spectra database to an effort with internationally approved reference stocks.

## Materials and Methods

Tubes and tips were purchased from Eppendorf. All chemicals were of highest purity (HPLC-grade) and purchased from Sigma-Aldrich.

The *Erwinia* strains came from various strain collections, others (described in ref. 3) and the *E. coli* K12-strains 1100 and W3350 were obtained from the collection of the JKI Dossenheim. The bacteria used for the reference database and the dendrogram were the following: *E. amylovora* CFBP1232 (T), *E. amylovora* Ea1/79 DSM 17948, *E. amylovora* 273 ATCC 49946, *E. amylovora* IL6 (rubus) (Lab collection JKI Dossenheim, isolated in Illinois, USA), *E. amylovora* MR1 (rubus) (Lab collection JKI Dossenheim, isolated in Michigan, USA), *E. pyrifoliae* 16/96 (T) DSM 12163, *E. pyrifoliae* 1/96 DSM 12162, *E. pyrifoliae* Ejp557 (Japan) (Lab collection JKI Dossenheim, isolated from Nashi pear, Hokkaido, Japan, 1994, A. Tanii), *E. tasmaniensis* 1/99 (T) DSM 17950, E. *tasmaniensis* 2/99 DSM 17949, *E. billingiae* Eb 660 (T) NCPPB660 and Eb 661 (T) NCPPB661, *E. persicina* CFBP3622 (T), *E. rhapontici* CFBP3618 (T), *E. psidii* CFBP3627 (T), Pectobacterium *cacticida* CFBP3628 (T), Brenneria *quercini* CFBP3617 (T), *E. mallotivora* CFBP2503 (T), *E. toletana* CFBP6631 (T), *E. papayae* CFBP5189 (T), E. *tracheiphila* CFBP2355 (T), *E. coli* 1100 (E. coli/ K-12, Lab collection JKI Dossenheim), *E. coli* W3350 (*E. coli*/ K-12, Lab collection JKI Dossenheim). *Erwinia* type strains are indicated (T); CFBP = Collection Française des Bactéries Phytopathogènes; DSMZ = German Collection of Microorganisms and Cell Cultures; NCPPB = National Collection of Plant Pathogenic Bacteria (UK); ATCC = American Type Culture Collection; JKI = Julius Kuehn Institute.

Infection of *in vitro* pear plants (micro-propagated plants): Pear leaves were wounded and inoculated with cells of the German *E*. *amylovora* strain Ea1/79. After incubating for 5 days, the infected pear plantlets displayed symptoms typical of fire blight infection, such as water soaking and necrosis accompanied by the production of bacterial ooze. We washed the bacteria from the plant surface with 1.5 ml water, centrifuged the samples at 1000× g for a minute, and decanted the liquid. Afterwards we suspended and inactivated the bacteria as is described below.

Isolation of *Erwinia* spp. from necrotic wood of pear trees (from Carinthia): Fifty milligram of dark bark slices contaminated with bacteria were immersed in 1 ml water. After soaking for 15 minutes, samples were diluted, and 200 µl of that were plated on LB agar with cycloheximide (50 µg/ml). White colonies were assayed on semi-selective agar for *E. amylovora* by using PCR and DNA sequencing at the JKI Dossenheim. *E. amylovora* colonies were processed as blind samples for MALDI analysis at the Max-Planck-Institute for Molecular Genetics (Berlin).

Cell culturing on agar: For cell culturing on agar plates, all dilutions were incubated for 2 days at 28°C. Bacteria were suspended from cell lawns in 1 ml water to a density of approximately 1 (light absorption at 600 nm) and centrifuged. The pellets were washed with 1 ml water and then the liquid was discarded. The presence of culture medium adhering to the bacterial colonies cells from agar had no visible effect on the mass signal patterns. The bacteria were inactivated as described below.

Culturing in liquid media: Bacteria grown on agar were inoculated into LB liquid medium with 1% glucose for the generation of reference spectra and in many cases for identification of unknown samples. The medium was autoclaved and then filtrated through a 0.2 µm nitrocellulose filter to remove particles. Replacement of LB-glucose by LB-glycerol showed little effect in the peak pattern distribution. Identification of bacteria grown on different media was reliably achieved as shown in Table 1. Once the bacteria have entered the stationary phase, the method is robust against growth times. However, other (minimal) media might have a stronger influence on the mass peak patterns. For the generation of reference spectra, we used LB-glucose as standard medium because most *Erwinia* bacteria grew well in this medium and resulted in very good mass spectra in terms of sensitivity and resolution.

Inactivation of bacteria: The bacteria were suspended in 300 µl water and inactivated by the addition of 800 µl ethanol at room temperature. The samples could be stored at room temperature for several days or at 4–8°C for several weeks. To assay for viability, we applied dilutions to agar plates and found no surviving *E. amylovora* cells already after an hour of storage in ethanol.

Protein extraction: This step was performed at room temperature. The solution was centrifuged at 25,000×g for 2 minutes and the supernatant was discarded. Again, centrifugation was performed for 2 minutes at 25,000×g and residual supernatant was discarded. Five to 20 µl of 70% formic acid were added to the “pellet” (1 to 5 mg, or less bacterial material), and mixed to re-suspend the bacteria. Then 5–20 µl acetonitrile were added, accordingly, and the sample was mixed carefully. The solution was centrifuged at 25,000×g for 2 minutes. The supernatant (∼5–20 µl) was transferred to a new tube immediately.

MALDI preparation: This step was performed at room temperature and at 20–80% air humidity. One microliter of the supernatant was placed onto a stainless steel target plate and led dry in air. Then, 1 µl of matrix (3 mg/ml solution of alpha-cyano-4-hydroxycinnamic acid in 50% acetonitrile/2.5% trifluor acetic acid) was overlaid onto the dried sample and led dry in air. This simple preparation method provided homogenous samples to enable automated measurements and sufficiently reproducible mass spectra. To increase data reliability, we applied each bacterial sample six times onto the target plate.

Mass spectrometry detection: Mass spectra were acquired using an Ultraflex I MALDI-TOF mass spectrometer (Bruker Daltonics, Bremen, Germany). Alternatively, a simpler MALDI-TOF instrument such as the benchtop Microflex (Bruker Daltonics) can be used without loosing data quality. We performed measurements in linear positive ion detection mode, using a Nd:YAG laser at maximum frequency of 66 Hz. Pulsed ion extraction (PIE) was set to zero. Acceleration voltage (IS1) was set to 20 kV. The mass range of spectra was from 2,000 to 20,000 m/z. The final resolution in the mass range of 7,000–10,000 m/z was optimized to be higher than 600 and absolute signal intensities were about 10^3^. Automated spectrum acquisition was performed using the Auto Execute software with fuzzy control of laser intensity. At least 10^7^ bacterial cells were required for high quality mass spectra. For reference spectra we measured six spots on the MALDI target. On each spot, four spectra with 10 times 100 laser shots were accumulated. Twenty spectra were stored for the reference spectra library. For identification we generally acquired spectra by accumulating 1000 laser shots in ten 100 shot portions.

Factors influencing the intensities of signal peaks comprise concentration and location of proteins in the bacterial cell and biophysical properties of proteins such as solubility, hydrophobicity, basicity, and compatibility with MALDI. In general, most of the proteins detected by MALDI protein bacterial profiling derive from highly abundant, basic ribosomal proteins [11].

Data analysis: Mass spectra were analyzed with Flex Analysis software 2.4 (Bruker Daltonics). Further bacterial data analysis was performed by software developed and tested by us that we termed BioTyper. The mass spectral input data can be listed in generic data formats such as the extensible markup language (XML) to make them independent from the hardware used. Spectra were pre-processed using default parameters for reference spectra libraries that we call main spectra libraries (MSPs). A maximum of 100 peaks with a signal-to-noise (S/N) ratio of 3 were selected in the range of 3,000–15,000 Da. Afterwards the main spectra were generated as a reference using all spectra given for a single microorganism. In general, 75 peaks were picked automatically, which occurred in at least 25% of the spectra and with a mass deviation of 200 ppm.

For the evaluation of mass spectra reproducibility, we loaded the spectra into the ClinProTools 2.1 software (Bruker Daltonics). Through this process mass spectra were firstly normalized before we applied baseline subtraction, peak detection, realignment, and peak-area calculation. The optimal settings resulted in an S/N ratio of 5, a Top Hat baseline subtraction with 10% as the minimal baseline width, and a 3-cycle Savitsky-Golay smoothing with a 10 Da-peak width filter. For the example shown in Figure 3 the coefficient of variation (CV) of each of the individual peak areas was determined; 100 peaks were taken for intra run assessment detected in 18 measurements and 75 peaks for inter run detected in 5 biological replicates. The mean CV for all of the signals from the same replicate sample was calculated to provide a measure of intra- and inter-run reproducibility.

Using the bacterial analysis software (Biotyper) and taking a list of mass signals and their intensities into consideration, dendrograms were generated by similarity scoring of a set of mass spectra. Dendrograms shown in this article had graphical distance values between species constructed from their reference spectra. A correlation function was used for calculating distance values. For graphical correlations an average statistical algorithm was applied as implemented in our software package. The maximal number of top level nodes was set to 2. As mentioned in the Figure legend 1, the arrangement of spectra on the left site of the dendrogram is arbitrary. Species with distance levels under 500 are reliably classified. DNA-based phylogenetic analysis (Figure 2) was done using the Mega (Molecular Evolutionary Genetics Analysis) program, version 3.1 (http://www.megasoftware.net/) [15].

The complete set of reference spectra compiled in the database of our software package is linked to the NCBI taxonomy database (http://www.ncbi.nlm.nih.gov/Taxonomy/).

For identity scoring, the algorithm implemented in the Biotyper software counted mass signals in experimental spectra that matched with reference spectra and vice versa. Furthermore, the algorithm applied correlates signal intensities of matched signals. Together, three scores obtained from such a procedure are multiplied and normalized to a value of 1000 and then converted in its common logarithm (3). Log scores over 2 indicated a reliable identification of species; log scores over 1.7 generally meant a reliable identification of bacterial genera. Log scores of 3 were obtained when spectra matched with themselves. For the identification of bacterial species, this pattern matching algorithm was routinely applied. For the distinction of highly similar mass spectra of closely related sub species, we used a weighted pattern matching algorithm. In practice, we assigned additional values to informative mass signals that were found in the reference spectra of these sub species. For the application of weighted pattern matching we used the masses and settings listed in Figure 5 and in Table 3. For more details on the BioTyper software the reader is referred to a handbook that is available from the authors as a hardcopy or an electronic version (CD of the complete analysis software package that is freely available for reproducing the results of this study and for testing the procedure shown in this article for additional bacterial genera).

SNP genotyping: Approximately 5 mg of bacterial pellet was re-suspended in 1 ml 0.1% Tween-20 and heated up to 65°C for 15 min. One micoliter was used as template for subsequent PCR. PCR was carried out in 10 µl volume. The PCR buffer consisted of 20 mM (NH_4_)_2_SO_4_, 75 mM Tris-HCl (pH 9.0), 0.01% Tween-20, 2.5 mM MgCl_2_, 0.5 M betaine solution, 0.3 mM dNTPs, 1 U conventional Taq polymerase (produced in-house), 0.025 U proofreading Taq polymerase (produced in-house), 0.3 µM forward primer (5′-CGATGACGTGGTGATACTGG-3′), 0.3 µM reverse primer (5′-TCGACTCCCCTACAGCCTTA-3′). After denaturating the PCR samples for 5 minutes at 95°C, amplifications were carried out at 94°C for 30 seconds, 65°C for 30 seconds, and 72°C for 30 seconds for 35 cycles. Finally, the samples were incubated at 72°C for 5 minutes. SNP genotypes were detected by mass spectrometry with the standard GOOD assay in negative ion mode [13], [16] as described in full detail in ref. 13 by using 2 µl of the PCR products generated from 10 µl reactions. The extension primer used for the GOOD assay was 5′-GCGACTTTCTTCGAAGGGG*AC-3′ (* indicates a phosphorothioate linkage). The reference sequences of the *galE* gene of two *E. amylovora* strains are shown in Figure S1.

## Supporting Information

Figure S1 Reference sequences. A SNP in the gal-E gene was used to differentiate E. amylovora strains of European/Mediterranean (Ea1/79) from American origin (Ea273).
(0.02 MB DOC)
