# Supplementary material for: Classification and Identification of Bacteria by Mass Spectrometry and Computational Analysis
Source: PLoS One. 2008 Jul 30;3(7):e2843. doi: 10.1371/journal.pone.0002843 (PMC2475672; doi:10.1371/journal.pone.0002843)
Supplement: Figure S1 — Reference sequences. A SNP in the gal-E gene was used to differentiate E. amylovora strains of European/Mediterranean (Ea1/79) from American origin (Ea273). (0.02 MB DOC) [file pone.0002843.s001.doc]

Alignment of galE-genes from Ea273 (Sanger) and Ea1/79 (M. Kube)

galE-Ea273 1 atgtctattttagtcacggggggagcaggctatattggatcccataccgt

galE-Ea1MK 1 ..................................................

galE-Ea273 51 actctcattgctgcaacgtggcgatgacgtggtgatactggataatctga

galE-Ea1MK 51 ..................................................

galE-Ea273 101 gtaatgcatcgcgcgagtcaattaatcgtgttgaaaagctaaccggtaaa

galE-Ea1MK 101 ..................................................

galE-Ea273 151 acggcgactttcttcgaaggggac**a**tcctcgatcgtagctgtttgcggag

galE-Ea1MK 151 ........................**c**.........................

galE-Ea273 201 tgttttcagcgctcaccgtatcagcgcagtgatccactttgccggactta

galE-Ea1MK 201 ..................................................

galE-Ea273 251 aggctgtaggggagtcgacgcgcaaaccgctggagtattaccaaaataat

galE-Ea1MK 251 ..................................................

galE-Ea273 301 gtcaccggtacgctggtactgctggaagagatgcgcagtgcaggggtgaa

galE-Ea1MK 301 ..................................................

galE-Ea273 351 tcaatttattttcagctcgtctgccaccgtatatggagccgatgcgccag

galE-Ea1MK 351 ..................................................

galE-Ea273 401 taccttatgttgaaaccacgccaattggcggtaccaccagcccctacggc

galE-Ea1MK 401 ..................................................

galE-Ea273 451 acgtctaagctgatggttgaacagattttacgtgactacgccaaagctaa

galE-Ea1MK 451 ..................................................

galE-Ea273 501 ccctgaattcaaaactattgcattacgctattttaatccggtcggtgctc

galE-Ea1MK 501 ..................................................

galE-Ea273 551 atgaatccggtcagattggcgaagatcctaacggtatcccaaacaacctg

galE-Ea1MK 551 ..................................................

galE-Ea273 601 ctaccgtatattgctcaggtcgccatcggtcggctggaaaaactcggcat

galE-Ea1MK 601 ..................................................

galE-Ea273 651 attcggtgatgactatccgactgaggatggcaccggcgtgcgggactaca

galE-Ea1MK 651 ..................................................

galE-Ea273 701 tccatgtgatggatctggcagagggacacctcaaggcactggatcatctt

galE-Ea1MK 701 ..................................................

galE-Ea273 751 tcggcgattgaaggttataaagcctataatcttggcgcggggaaaggcta

galE-Ea1MK 751 ..................................................

galE-Ea273 801 ctcggtgctggaaatggttaaggcgtttgaaaaagcgtctggccgcacgg

galE-Ea1MK 801 ..................................................

galE-Ea273 851 tcgcatatcagatttctccacgtcgtgatggcgacctggctgctttctgg

galE-Ea1MK 851 ..................................................

galE-Ea273 901 gccgatgccacgctggctgataaagagttgaactggcgcgtctcgcgcgg

galE-Ea1MK 901 ..................................................

galE-Ea273 951 tattgacgagatgatgcgtgatacatggaactggcagagccagaatcccc

galE-Ea1MK 951 ..................................................

galE-Ea273 1001 aaggctatagttga

galE-Ea1MK 1001 ..............
